# Migratory Birds, Ticks, and Crimean-Congo Hemorrhagic Fever Virus

**DOI:** 10.3201/eid1812.120718

**Published:** 2012-12

**Authors:** Mats Lindeborg, Christos Barboutis, Christian Ehrenborg, Thord Fransson, Thomas G.T. Jaenson, Per-Eric Lindgren, Åke Lundkvist, Fredrik Nyström, Erik Salaneck, Jonas Waldenström, Björn Olsen

**Affiliations:** Uppsala University, Uppsala, Sweden (M. Lindeborg, C. Ehrenborg, T.G.T. Jaenson, E. Salaneck, B. Olsen);; Hellenic Ornithological Society and Natural History Museum of Crete, Crete, Greece (C. Barboutis);; Swedish Museum of Natural History, Stockholm, Sweden (T. Fransson);; Linköping University, Linköping, Sweden (P.-E. Lindgren, F. Nyström);; Swedish Institute for Infectious Disease Control, Solna, Sweden (A. Lundkvist);; and Linnaeus University, Kalmar, Sweden (J. Waldenström)

**Keywords:** Crimean-Congo hemorrhagic fever virus, ticks, tick-borne diseases, animal migration, zoonoses, migratory birds, vector-borne infections, Europe, Africa, Crimean-Congo hemorrhagic fever

**To the Editor:** In a recently published study, Estrada-Peña et al. reported the finding of Crimean-Congo hemorrhagic fever virus (CCHFV) in adult *Hyalomma lusitanicum* ticks from red deer (*Cervus elaphus*) in Spain during 2010 (*1*). Phylogenetic analysis showed that the virus was most likely of African origin. Here, we present a model for the transfer of CCHFV-infected ticks by migratory birds from Africa to Europe.

CCHFV is an RNA virus in the genus *Nairovirus*, family *Bunyaviridae*. It is transmitted to humans through tick bites or by contact with blood or tissues from infected ticks, livestock, or humans. Manifestations of severe cases are internal and external hemorrhages and multiorgan failure; the case-fatality rate is ≈30% (*2*,*3*). CCHFV has the widest geographic distribution of any tick-borne virus, encompassing ≈30 countries from eastern China through Asia, the Middle East, and southeastern Europe to Africa (*3*,*4*). During the past decade, the virus has emerged in new areas of Europe, Africa, the Middle East, and Asia and has increased in disease-endemic areas (*5*) (Technical Appendix).

In response to the emergence of CCHFV in Europe, during spring 2009 and 2010, we screened migratory birds for ticks as they traveled from Africa to Europe. At 2 bird observatories on the Mediterranean Sea (Capri, Italy, and Antikythira, Greece), 14,824 birds of 78 different species were caught and examined for ticks. Most (88%) of the 747 collected ticks were identified as members of the *Hyalomma marginatum* complex, most probably *H. rufipes* and *H. marginatum* sensu stricto (s.s.), i.e., the principal vectors of CCHFV (*2*). Of 10 morphologically representative ticks, 9 were identified by molecular methods as *H. rufipes* and 1 as *H. marginatum* s.s (*6*).

Ticks belonging to the *H. marginatum* complex are common in large parts of the African and Eurasian continents. The immature ticks feed mainly on birds and, to a lesser extent, on small mammals, whereas the adults actively seek larger mammals, including hares, wild and domesticated ungulates, or humans (*4*). In accordance with this pattern, 99% of the collected ticks in our study were larvae and nymphs.

On April 23, 2009, a woodchat shrike (*Lanius senator senator*) was caught at the Antikythira Bird Observatory in the Greek archipelago. The bird was a female in her second calendar year and harbored 19 *H. marginatum* complex ticks (3 larvae and 16 nymphs, most likely *H. rufipes*). Three of the nymphs, 1 half-fed and 2 fully engorged, were found positive by real-time PCR for the CCHFV small (S) segment by using methods previously described (*7*), amplifying a 127-bp product. The 3 positive samples were sequenced and found to be identical. Previous studies, based on the S segments, have identified 7 phylogenetically distinct genotypes: Africa 1–3, Asia 1–2, and Europe 1–2 (*8*). Europe 1 has been reported from Russia, Turkey, Greece, Bulgaria, and the Balkans, and Europe 2 is the nonpathogenic strain AP92 found in Greece. Alignment of the Antikythira strain with CCHFV S segment sequences deposited in GenBank showed that it had the greatest similarity with strains belonging to the genotype Africa 3 (*8*). In addition, a phylogenetic tree clearly places the Antikythira sequence within the Africa 3 clade (Figure).

**Figure:**
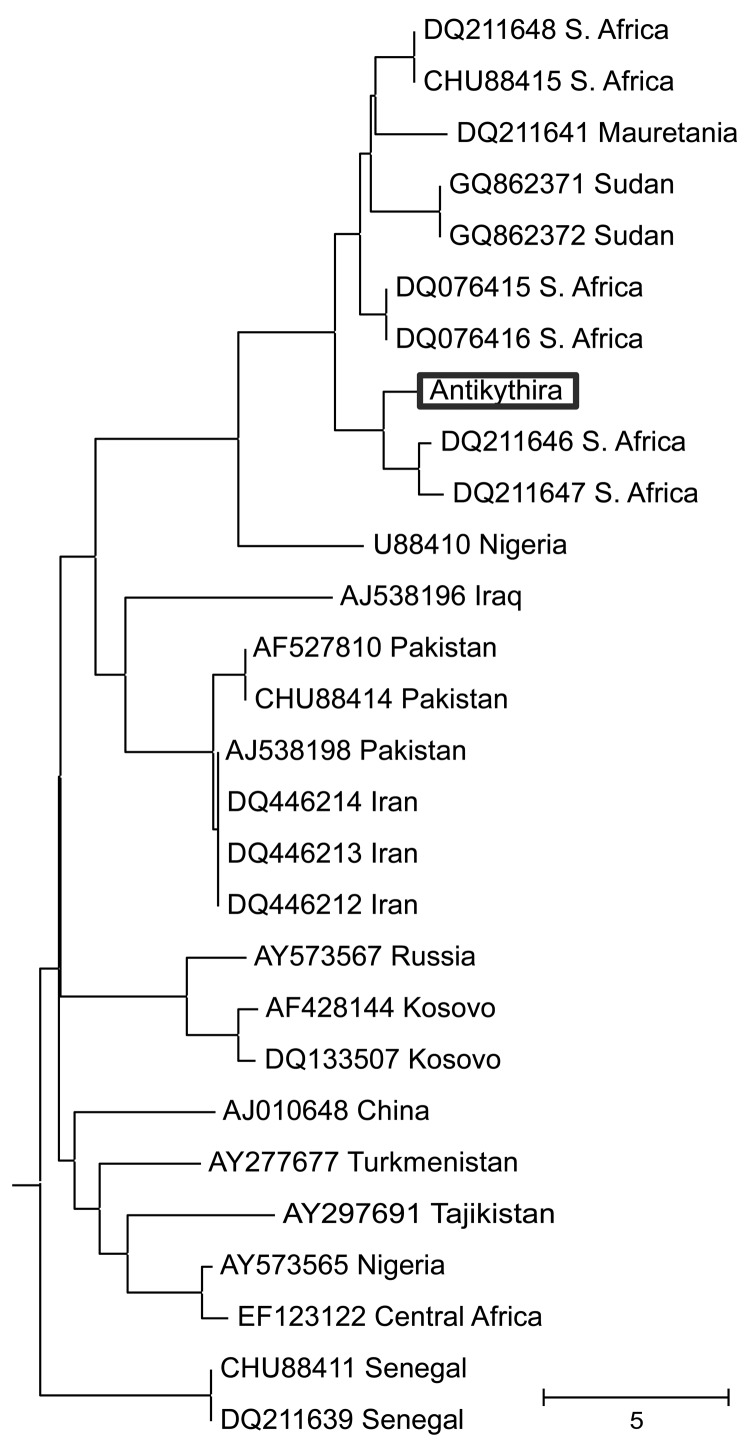
Neighbor-joining tree of Crimean-Congo hemorrhagic fever virus small segment sequences retrieved from GenBank and the novel 127-bp sequence isolated in this study (boxed). The tree is drawn to scale, with branch lengths in the same units as those of the evolutionary distances used to infer the phylogenetic tree. A total of 127 positions were in the final dataset. Trees generated with maximum-likelihood and maximum-parsimony methods (not shown) exhibited nearly identical topology to this tree. The corresponding part of the Nairobi sheep disease virus small segment was used as an outgroup. The analyses were conducted in MEGA5 software (www.megasoftware.net) using a ClustalW alignment. Accession numbers and geographic origins of the sequences are shown. Scale bar indicates number of base differences per sequence. S. Africa, South Africa.

The woodchat shrike winters in a belt from Senegal to Somalia and breeds in southern Europe and northern Africa (*9*). The Antikythira bird was caught during its rapid northward migration on a small island where birds normally stop over briefly just after crossing the Sahara Desert and the Mediterranean Sea. Also, the infected ticks were either half fed or fully engorged nymphs that, in the case of *H. marginatum* complex ticks, normally attach to the bird as larvae; this finding indicates that these ticks had attached before the bird began migration. Furthermore, 9/10 morphologically representative ticks were identified by molecular methods as *H. rufipes*, a species within the *H. marginatum* complex most common on the African continent (*4*,*6*). On the basis of these findings, we propose that this bird was infested somewhere in sub-Saharan Africa.

Migratory birds acting as long-distance transporters of ticks containing various human pathogens have been reported (*10*). Pre-adult ticks can stay attached to avian hosts during migration, thereafter detaching at breeding or stopover sites, where mammalian hosts can potentially establish new foci (*4*). Regarding the finding in Spain (*1*), one could speculate that new cycles of CCHFV transmission could be initiated through viremic or nonviremic (cofeeding) mechanisms involving, for example, transstadially infected adult *H. rufipes* ticks and susceptible *H. lusitanicum* ticks that are feeding on the same mammalian host*.*

Further research is needed on the interaction between birds and ticks in relation to the geographic distribution of CCHFV. Monitoring the influx of migratory birds carrying CCHFV-infected ticks might give disease-prevention authorities a useful tool for predicting the potential emergence of new disease foci in Europe.

Technical Appendix Map showing emergence and reemergence of Crimean-Congo hemorrhagic fever in Africa since 2005.
