# Supplementary material for: Migratory Birds, Ticks, and Crimean-Congo Hemorrhagic Fever Virus
Source: Emerg Infect Dis. 2012 Dec;18(12):2095–7. doi: 10.3201/eid1812.120718 (PMC3557898; doi:10.3201/eid1812.120718)
Supplement: Technical Appendix — Map showing emergence and reemergence of Crimean-Congo hemorrhagic fever in Africa since 2005. [file 12-0718-Techapp-s1.pdf]

Article DOI: http://dx.doi.org/10.3201/eid1812.120718

Migratory Birds, Ticks, and Crimean-Congo Hemorrhagic Fever Virus

Technical Appendix

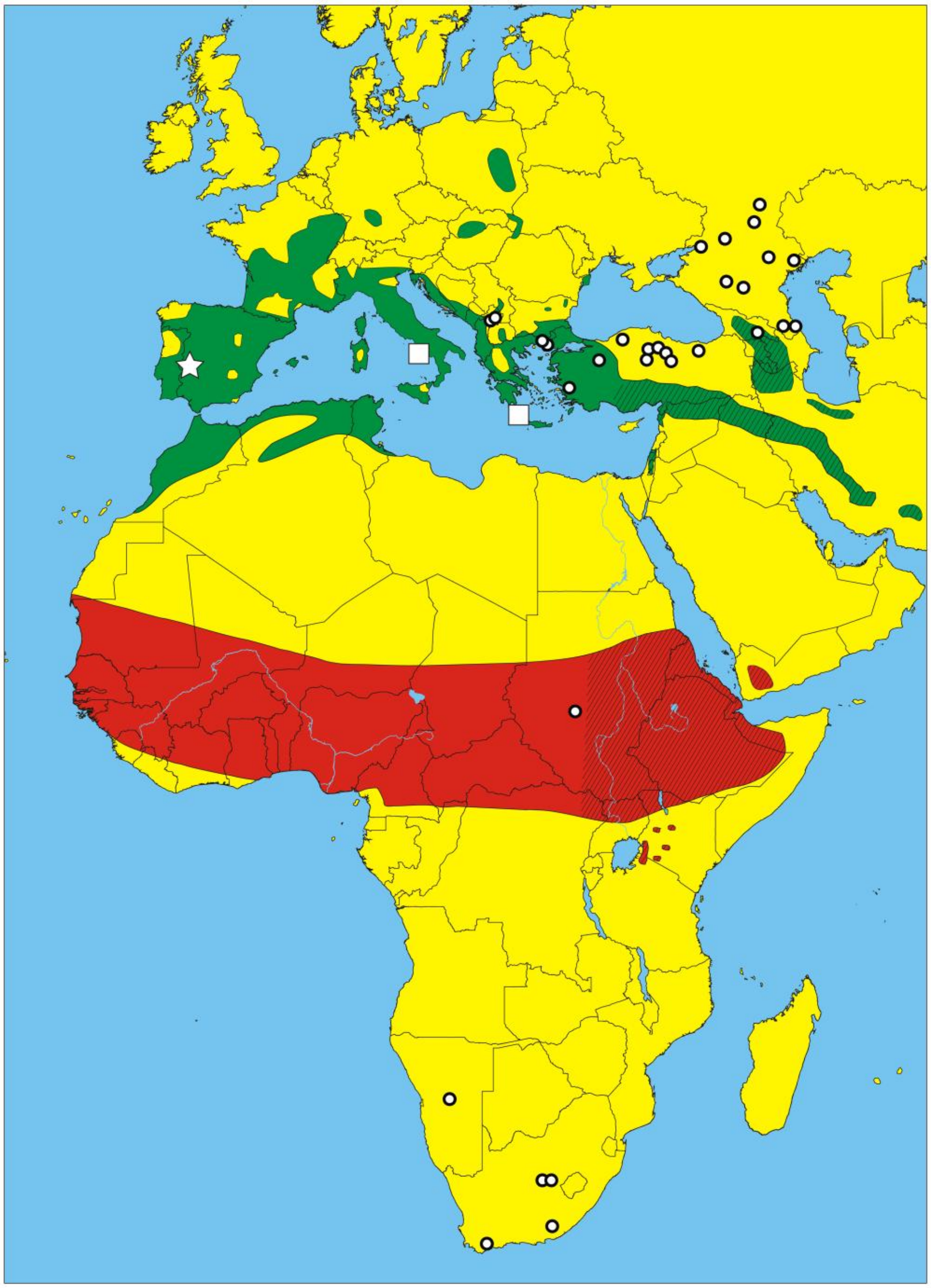

Technical Appendix Figure. Occurrence of Crimean-Congo hemorrhagic fever in Africa and Eurasia since 2005. Each circle represents an outbreak reported by Pro-MED Mail (www.promedmail.org). Red and green indicate wintering and breeding areas, respectively, for the woodchat shrike (*Lanius senator senator*); hatched area represents the subspecies *L.s. niloticus*. Squares indicate the 2 bird observatories used; star indicates record of Crimean-Congo hemorrhagic fever virus–infected *Hyalomma lusitanicum* in Spain (*1*). Illustration by Thord Fransson.

Reference

1. Estrada-Peña A, Palomar AN, Santibáñez P, Sánchez N, Habela MA, Portillo A, et al. Crimean-Congo hemorrhagic fever virus in ticks, southwestern Europe, 2010. Emerg Infect Dis. 2012;18:179–80. PubMed http://dx.doi.org/10.3201/eid1801.111040
